# XGBoost-Based Remaining Useful Life Estimation Model with Extended Kalman Particle Filter for Lithium-Ion Batteries

**DOI:** 10.3390/s22239522

**Published:** 2022-12-06

**Authors:** Sadiqa Jafari, Yung-Cheol Byun

**Affiliations:** 1 Department of Electronic Engineering, Institute of Information Science & Technology, Jeju National University, Jeju 63243, Republic of Korea; 2 Department of Computer Engineering, Major of Electronic Engineering, Institute of Information Science & Technology, Jeju National University, Jeju 63243, Republic of Korea

**Keywords:** lithium-ion battery, remaining useful life, XGBoost, particle filter

## Abstract

The instability and variable lifetime are the benefits of high efficiency and low-cost issues in lithium-ion batteries.An accurate equipment’s remaining useful life prediction is essential for successful requirement-based maintenance to improve dependability and lower total maintenance costs. However, it is challenging to assess a battery’s working capacity, and specific prediction methods are unable to represent the uncertainty. A scientific evaluation and prediction of a lithium-ion battery’s state of health (SOH), mainly its remaining useful life (RUL), is crucial to ensuring the battery’s safety and dependability over its entire life cycle and preventing as many catastrophic accidents as feasible. Many strategies have been developed to determine the prediction of the RUL and SOH of lithium-ion batteries, including particle filters (PFs). This paper develops a novel PF-based technique for lithium-ion battery RUL estimation, combining a Kalman filter (KF) with a PF to analyze battery operating data. The PF method is used as the core, and extreme gradient boosting (XGBoost) is used as the observation RUL battery prediction. Due to the powerful nonlinear fitting capabilities, XGBoost is used to map the connection between the retrieved features and the RUL. The life cycle testing aims to gather precise and trustworthy data for RUL prediction. RUL prediction results demonstrate the improved accuracy of our suggested strategy compared to that of other methods. The experiment findings show that the suggested technique can increase the accuracy of RUL prediction when applied to a lithium-ion battery’s cycle life data set. The results demonstrate the benefit of the presented method in achieving a more accurate remaining useful life prediction.

## 1. Introduction

Lithium-ion batteries are presently the primary source of electricity for electric vehicles (EVs). Utilizing lithium-ion batteries has several advantages over conventional NiMH or lead–acid batteries, including a higher energy density and nominal voltage, as well as lower self-discharge rates and a longer lifetime. The use of lithium-ion batteries has been widespread in many industrial applications. Battery failure can have serious consequences, such as degraded performance or functional impairment [1,2]. The lithium-ion battery packs in EVs are comprised of hundreds to thousands of cells connected in series, in parallel, or even more complicated ways to provide the operating power and energy requirements. Therefore, it is essential to ensure and monitor the proper operation of the battery pack [3]. Battery power and capacity decrease and inevitably decline with age battery, affecting the performance and service life and potentially creating certain safety risks for batteries. Battery deterioration occurs under all circumstances, and the increase in battery life reduces the costs and environmental responsibilities associated with manufacturing new batteries. These are sustainable approaches for managing battery resources and avoiding degradation [4,5]. The development of a degradation model is necessary from the standpoint of battery operation; degradation can affect the parameters of the battery, such as health and life; thus, lithium-ion battery life can be increased by comprehending the functioning basics and modeling the batteries’ degradation [6].

Using a battery management system is a well-known method for keeping track of a battery pack’s condition and ensuring its security and performance improvement (BMS). The BMS’s ability to determine the battery’s state of charge (SOC) and state of health (SOH) is one of its key capabilities [7]. The SOH refers to the battery’s physical state compared to a brand-new battery. In addition, the SOC is frequently explained as the ratio of the remaining capacity over the rated value. An alternative for the SOH is battery the RUL, defined as the discrepancy between the total and actual cycle numbers. A lithium-ion battery’s RUL is typically referred to as its remaining life. The threshold for retiring batteries in an electric car is 80% capacity. Repeatedly carrying out charge and discharge cycle studies has been a frequent technique. However, this approach requires extensive testing, is expensive, and can also have irreparable adverse effects. Therefore, it is crucial to accurately anticipate the RUL of lithium-ion batteries in a short time. In reality, the SOH and RUL are two primary indications that when lithium-ion batteries age and are subject to the effects of working circumstances, the battery’s performance will steadily decline over time. The overall usable capacity loss and resistance increase, which together make up the SOH, are indicators of the battery’s aging state. The RUL stands for the duration from the present to the battery’s end of usable life. In recent years, a number of methodologies have been presented for the RUL prediction of lithium-ion batteries. The RUL prediction depends on an accurate SOH estimation, which can be obtained using direct measurement techniques, model-based strategies, and data-driven techniques [8].

**Figure 1:**
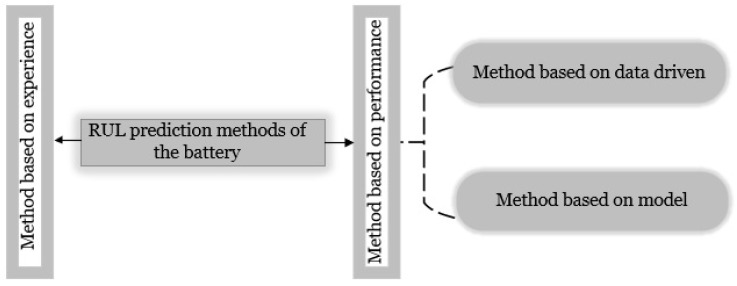
Classification of approaches for predicting remaining useful life.

Batteries have inconsistent regeneration along with nonlinear deterioration. Battery parameters other than current capacity must be considered to predict the battery’s RUL accurately. Additionally, the battery generates power through an electrochemical process, and when the battery is continually charged and discharged, the overall available capacity diminishes. In other words, repeated chemical reactions reduce the active material, affecting the output power and the battery’s capacity, which lowers performance [9,10]. Figure 1 illustrates how strategies for RUL prediction may be categorized into two groups: methods based on performance and techniques based on experience. The experience-based approach is a statistical technique that operates many test data and uses long-term experience to accurately and forecast typical battery life. It is easy to see how this method’s reliance on knowledge, especially in a dynamic and changing environment, results in a lack of adaptability.

The performance prediction technique, a sort of indirect prediction approach, consists of two stages: detecting the battery’s deterioration condition and predicting performance. The method based on a data-driven mode is also part of the performance-based procedure. Three parameters—charge-discharge behavior, capacity, and internal resistance—are frequently used to measure the SOH, serving as the primary indicator of the battery’s life cycle. There are two categories of methods to estimate the SOH, model-based and data-driven [11,12]. Due to the abundance of battery data and improved computational capabilities, machine learning (ML) has lately become one of the data-driven methodologies that are most effective for SOH and RUL estimates and modeling battery degradation [13]. Multiple ML models have been used in various studies to give a complete picture of ML-based RUL estimation, depending on the data properties, input features, target variables, experimental settings, battery design, and prediction accuracy. The RUL prediction calculates a system’s remaining usable life, which is crucial for planning maintenance and mitigating contingencies. Much research has been documented on creating predictive models that can forecast a system’s RUL. These models can be broadly categorized into three groups: models based on experience, models based on data, and models based on physics. However, there is no ideal method for estimating the RUL due to the system’s complexity, data accessibility, and application limitations. The two main components of the model-driven technique are the empirical model and KF methods. These techniques were explicitly focused on developing hybrid predictive approaches, attempting to take advantage of the benefits of combining the predictive models in the various aforementioned categories for RUL prediction [14]. The empirical model, which includes exponential, linear, polynomial, and Verhulst models, focuses on determining the underlying mathematical connection of the capacity degradation trajectory. The empirical model can predict future life trajectories in addition to the RUL [15]. However, producing an accurate RUL prediction is challenging due to the sensitivity of data fitting and sample variations [16].

A mathematical formula based on the input of the number of cycles and capacity output was established using data fitting to represent the battery deterioration state [17]. The KF approach uses the EKF, unscented KF, PF, and spherical volume KF based on the state estimate for RUL prediction [18]. By using observation data to update and correct the model in real time, the KF technique effectively addresses the nonlinear problem. Although the KF technique can improve the empirical model method’s convergence, temperature, current, and outside disturbances changes immediately influence the battery model’s correctness. As a result, building a reliable model for RUL prediction is challenging. Through analyzing monitoring data and discovering battery deterioration information, the data-driven approach divides the problem into a study of the mechanism and internal chemical reaction [19]. Accurately estimating a battery’s remaining useful life is advantageous for lithium-ion batteries because it improves safety and lengthens battery life. A hybrid model was built on an attention mechanism, and a bidirectional extended short-term memory network was suggested to improve prediction accuracy. This model could successfully predict using early data from the target battery [20,21]. If the battery is allowed to function, it will damage the equipment and cause battery deflagration, which will cause casualties. In order to avoid battery failure, it is essential to assess the battery’s working condition and replace it accurately. In order to ensure the equipment works safely and reliably, it is essential to manage the entire life cycle of each component. Therefore, it is important to thoroughly research the capacity attenuation and RUL prediction of lithium-ion batteries. This work presents a novel approach that integrates XGBoost and a PF to predict the RUL for the batteries. Battery current, terminal voltage, voltage change rate, and temperature change rate are also retrieved as features for the training dataset from the training layer. The RUL assesses battery dependability to predict when failure would occur and further reduce battery. XGBoost’s powerful nonlinear fitting capability made the connection between the extracted features possible. Additionally, this layer used PF to reduce measurement noise, enhancing the accuracy of XGBoost. The experimental findings on the discharge data set demonstrate that this approach is more accurate and stable in the XGBoost technique.

Our main contributions to the study are summarized in the following:

- Along with the discharge capacity degradation profile, the suggested approach considers input factors from the charging profiles, such as voltage, current, and temperature.
- XGBoost model uses the associated characteristics collected by the PF. technique as the reference input.
- The RUL estimate results are obtained using the XGBoost model.
- We conduct extensive experiments to show the changes in the estimation accuracy.
- The innovative aspect of the proposed study is the creation of a clever framework for a lithium-ion battery’s RUL prediction and the algorithm’s training using multiple dataset combinations made available by NASA. Moreover, in the paper’s final portion, we compare the proposed method with the recent work on the XGBoost model’s performance with other methods.

The rest of this paper is organized as follows: Section 2 describes the battery capacity degradation and its relationship with voltage, current, and temperature charging profiles. Section 3 describes the traditional RUL prediction and the proposed method. Section 4 introduces the dataset, and we present the learning process and model selection in Section 5 and the experimental results in Section 6. At the end of the paper, we provide the conclusion and further work.

## 2. Related Work

There are a wide variety of applications for battery energy storage systems (BESS), such as consumer electronics, electronic vehicles (EVs), uninterrupted power supplies (UPS), transportation, and military applications. Lithium-ion batteries have a maximum energy density and an extended life cycle; they are the best choice for EV applications. However, lithium-ion batteries’ chemical nature means they lose their capacity with repeated discharging and charging over their lifetime. As lithium-ion batteries age, their SOH decreases. Consequently, aging batteries should be assessed for safety and optimal performance. Battery cells or modules should be replaced from the battery system if the health status alarm reaches the preset limit. The SOH for lithium-ion batteries does not have a defined definition at the moment. The most commonly used indicators of a battery’s energy and power capability are the remaining capacity, the internal impedance, and the remaining life [22,23,24,25].

With the advancement of EVs, the Internet of things (IoT), and the electrification era, the performance of energy storage systems has become increasingly crucial. Among other energy storage options, the lithium-ion battery has a high energy density, high power density, and low self-discharge [26]. The battery management system technology also faces more challenging requirements. Several functions should be included in a comprehensive BMS. These include receiving battery data, determining and predicting the battery status, controlling charge and discharge, protection from static discharges, thermal management, balance control, and communication between the BMS and the battery [27,28,29]. The BMS performance is evaluated based on the accuracy of the state estimation. Energy storage systems can run reliably and last longer with high-quality BMSs [30]. Multitasking capabilities should be provided by the optimized BMS so that multiple tasks can work concurrently and smoothly. Also included is a real-time working system that monitors the system parameters and status, allowing the system to be adjusted in real time. Despite their long lifespans, lithium-ion batteries also have a limited lifetime. A wide range of applications cannot use lithium-ion batteries due to their high price and aging [31]. Equipment service life will be reduced, and battery aging will affect safety. A battery typically reaches the end of its useful life when its capacity falls to 80% of its initial value [32,33].

The RUL is described in the battery manufacturer’s specifications as the time between the current observation and the battery’s EoL [34]. In data-driven methods, machine learning uses human knowledge to enable functions and performance previously unavailable to machines while also allowing humans to interact with ML systems, making ML decisions more understandable [35,36]. When historical life-cycle data are available, ML methods should be used to predict the RUL [37]. Support vector machines (SVMs) have generated widespread interest due to their highly effective performance on small training data sets. An SVM is a nonparametric machine learning technique based on kernels. Training data sets increase in size as the number of support vectors increases. This method’s adaptability is increased by its capacity to be changed in accordance with the features of the system, which means it can give enough data support in complicated systems [38,39]. Based on the historical database, a DNN-based strategy was introduced; the target battery’s closest competitor was selected using the average Euclidean distance based (AED) method. Finally, a prediction model based on the stacked denoising autoencoder (SDA) was trained using data, and the RUL was calculated for the target battery. The prediction speed was increased by nearly 30% by improving the method in [40]. A new DNN prediction model predicted the RUL of lithium-ion batteries. Short-term measurements were used to complete RUL predictions utilizing an end-to-end deep learning framework. Lithium-ion battery’s RUL prediction was studied in [41] using DNNs, in which the extracted features were used to predict capacity, and two DNNs were trained to do so. In one DNN, the capacity attenuation of the impedance attenuation was analyzed statistically as the degree of degradation increased. In the other one, the probability prediction of the remaining service life was made based on the capacity attenuation trend [42]. Time series data are frequently processed using recurrent neural networks because of their time series memory. This model has a classic three-layer structure called an SLFNN. RNN inputs are based on time series variables according to time changes at each moment. RNNs can predict input variables through training [43,44]. By using the incremental capacity analysis approach, the RUL of the battery was projected based on the connection between the battery capacity and the remaining service lifetime in [45] based on the radial basis function NN model. It could estimate the RUL of lithium-ion batteries online by processing historical data sets. Feed-forward neural networks (FFNNs) were trained using the feature vector as input, and 40 neurons were used in the hidden layer [46]. A manufacturer of lithium-ion batteries has presented to create a battery capacity degradation model based on an exponential function and a nonlinear least squares method to increase prediction accuracy. In order to predict the RUL of lithium-ion batteries, this model was utilized [47]. Based on batteries, a strategy was proposed to predict the RUL, including capacity regeneration and random fluctuations [48].

A classification method was suggested as a gross estimation using support vector regression (SVR) to determine the accurate RUL, where the essential characteristics were taken from the voltage and temperature profiles after analyzing the cycling data of lithium-ion batteries under various operating situations. The multistage technique produced quicker calculations in addition to a better accuracy; therefore, a trained model was employed in real time [49]. Another paper offered a model based on low-frequency electrochemical impedance ranges that facilitated the time-domain matching circuit model for simulating dynamic battery characteristics from electrochemical and physical perspectives to predict and estimate battery state [50]. A paper studied a new long short-term memory method for the RUL estimation and prediction by combining a multilayer perception that applied the features of the sensor signal timing and obtained better prediction results [51]. The BMS for lithium-ion batteries in new energy vehicles generally includes a function for estimating the SOC. The conventional classification of SOC estimation methods was reviewed in one paper. The article focused on the online state of charge estimation methods for lithium-ion batteries in electric vehicles. The SOC estimation was the best performance known in the battery management system. A novel viewpoint was put forward that concentrated on a SOC estimate method error analysis based on different methods involving error flow charts to investigate SOC error sources [52]. Another paper proposed a fusion RUL prediction technique using a deep belief network (DBN) and a relevance vector machine (RVM). The lithium-ion battery’s RUL prediction was performed using the DBN’s extracted features, and the RUL prediction was supplied by the RVM, employing the extracted features [53].

The capacity to accurately anticipate the RUL of lithium-ion batteries is crucial, given the growing need for more stable and dependable electrical systems. Recurrent neural networks (RNNs) and some of their offshoots, including long short-term memory, have excelled at several sequential tasks. On the other hand, because of their iterative structure along the time axis, RNNs require a long time for information to flow through the network for prediction. The attention method allows the network to focus on specific sections of sequences while utilizing the parallelization advantages of CNN on GPUs, and positional encoding adds location information [54,55]. Employing various quantifiable data from the BMS, such as charging profiles with varying voltage, current, and temperature as they age, one may estimate the RUL even when capacity regeneration is present. For a LSTM prediction to determine the RUL of a battery, the input layer and output layer were matched as a one-to-one structure. Additionally, many-to-one designs were adaptable to various input sources, which allowed for a reduction in the number of parameters for improved generalization [56]. Different RUL prediction strategies were utilized in their work, with additional benefits. Table 1 summarizes the RUL prediction methods discussed above.

## 3. Methodology

**Figure 2:**
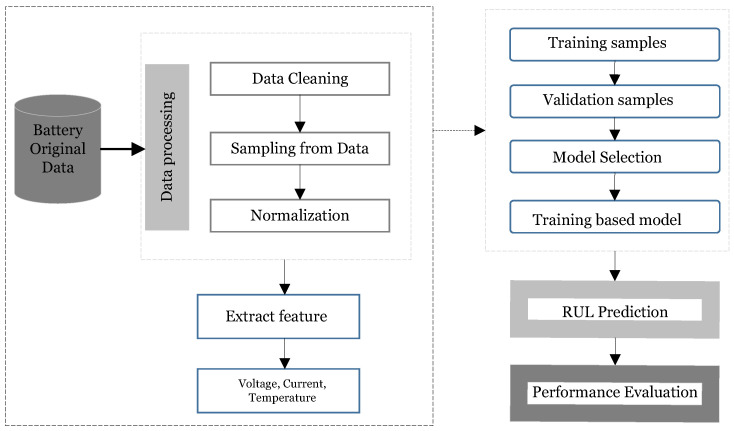
RUL prediction framework based on the proposed method for lithium-ion batteries.

This study addresses the erroneous RUL prediction, a random process with Markov properties that describes how a battery deteriorates. The battery’s use state at any time, including the load, temperature, and other factors, is independent of its initial state, and its use state at every subsequent time depends on the present state. As a result, the battery’s deterioration state complies with a first-order Markov model. The PF is the ideal solution for nonlinear issues that follow the Markov model because the battery’s internal chemical reaction is intricate and characteristic of a nonlinear system. The XGBoost algorithm is a concise representation of the prediction technique suggested in this research. To more accurately illustrate the RUL prediction technique, its flow is presented for a lithium-ion battery in Figure 2. Data preprocessing, training, and testing are the three processes in this system. Data preprocessing is used in the first phase to eliminate anomalous data and to minimize oscillation in small time intervals; we average the data oversampling period. After preprocessing, the characteristics are extracted from the original data. Figure 2 shows that we average the oversampling data interval in the first phase after eliminating anomalous data using data cleaning to prevent oscillation in brief time intervals. In order to eliminate instability in short time intervals after anomalous data are removed by data cleaning, we average the oversampling data interval in the first step. The data are then divided into training, validation, and test sets after being normalized using the min-max method. In the second stage, the model is chosen by determining the hyperparameters using cross-validation. In the third stage, the RUL prediction and progress performance evaluation is carried out using the model developed in the previous stage. The XGBoost model receives the recovered characteristics as inputs and outputs the anticipated RUL of the lithium-ion battery.

### 3.1. The Standard Theory of Particle Filtering

The PF algorithm, which can handle any nonlinear non-Gaussian issue, is a Bayesian state estimation approach based on the Monte Carlo sampling technique. It selects samples, also known as particles, from a posterior distribution using the Monte Carlo approach and gives each particle a weight [64,65]. A dynamic state-space model, further broken down into a state transition model and measurement model can be used to formulate many dynamic operations and physical systems in practice. A particle filter concentrates on a broader scenario where the system may be nonlinear, and the noise distribution may not be Gaussian, as opposed to the KF, which exclusively considers linear systems and Gaussian noise [66]. A particle filter concentrates on a more generic scenario where the system is nonlinear and the noise distribution is non-Gaussian. The dynamic system’s state equation and measurement equation are the ones shown below as step one:

(1) $$X_{z}=F\left(X_{z-1},G\right)\to z\epsilon N$$

(2) $$y_{z}=H\left(X_{z},V_{z}\right)\to z\epsilon N$$

In Equations (1) and (2), $X_{z}$ represents the state vector, $y_{z}$ is the measurement vector or the system outputs, *G* defines the system noise, and $V_{z}$ describes the measurement noise. The prior distribution is given by Equation (3) as step two:

(3) $$\alpha =\Phi (X_{0:z-1}^{i}|y_{1:z-1})$$

The approximation of the posterior distribution is shown in Equation (4) where n represents the posterior distribution, $W_{n}^{i}$ represents the sample weights, λ represents the Dirac function.

(4) $$\Phi \left(X_{0:z}^{i}|y_{1:z}\right)=\sum_{i=1}^{n}W_{z}^{i}\lambda \left(x_{0:z}-x_{0:z}^{i}\right)$$

According to Equation (4), $\sum_{i=1}^{Z}=W_{n}^{i}=1$ and direct sampling from a posterior distribution is challenging. As a result, we employed a different method called importance sampling, which selected samples from the importance distribution as in Equation (5) as step three:

(5) $$\sum_{i}^{n}\lambda \left(x_{0:z}-x_{0:z}^{i}\right)$$

As step 4, we derived Equation (6) representing the weight update as:

(6) $$W_{z}^{i}=W_{z-1}^{i}\beta$$

where β equals $\Phi \left(y_{z}|X_{z}\right)\Phi \left(y_{z}|X_{z-1}\right)/\gamma_{p}\left(X_{z}|X_{0:z},y_{1:z}\right)$, which represents the importance distribution.

### 3.2. Explanation of the Extended Kalman–PF Approach

**Figure 3:**
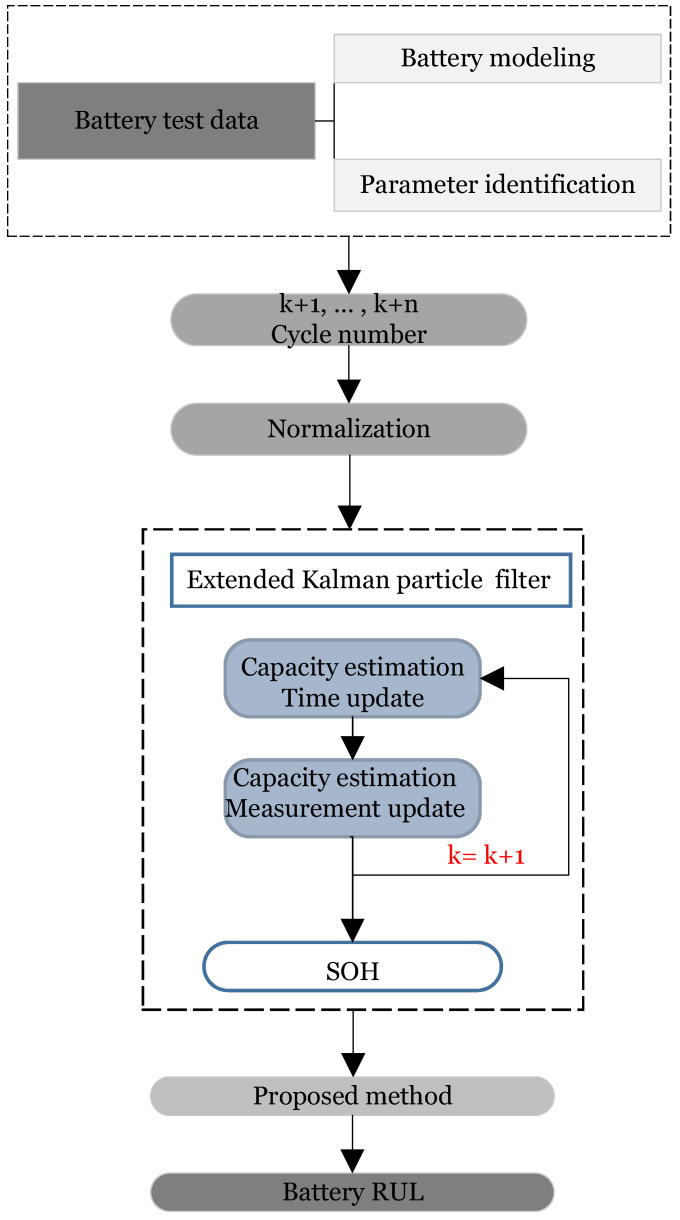
Flow diagram of EKPF algorithm.

Figure 3 depicts the flow diagram for the EKPF approach, which shows how the EKPF approach is used to choose particles iteratively and select a sufficient number of particles with higher weights by establishing a decision rule. With the help of this measure, the issue of an insufficient particle in the widely used PF algorithm can be effectively resolved, and particles with large weights become more dominant.

### 3.3. XGBoost Method

XGBoost is an effective classification, and regression algorithm [67]. Based on the gradient-boosting architecture, XGBoost continuously incorporates new decision trees to suit a value with several residual iterations and enhance the learners’ effectiveness. In contrast to gradient boosting, XGBoost operates a Taylor addition to compare the loss function. The model has a better trade-off between bias and variance, often requiring fewer decision trees to achieve higher accuracy. Here is an explanation of XGBoost.

XGBoost is used to create a mapping relationship between the extracted characteristics to estimate a battery’s RUL correctly. Finding a new suitable decision tree to match the residual error of the most recent prediction is the central concept of XGBoost. The anticipated value gets more accurate with each repetition. Depending on how each tree is built, various leaf nodes are assigned samples. The weights of the leaf nodes are added to determine a tree’s prediction score. It is possible to estimate the expected value of the data set by aggregating the scores for each tree. Consider a sample set with *n* samples for the features as in Equation (7):

(7) $$D=\left(X_{z},y_{z}\right)\to z=1,2,3,...,N$$

By employing *n* additive functions, a tree ensemble model is employed to estimate the expected value ${R\hat{U}L}_{z}$ in Equation (8):

(8) $${R\hat{U}L}_{z}=\sum_{1}^{n}F_{n}\left(X_{z}\right)\to F_{n}\epsilon \tau$$

where decision trees in the set τ are as follows:

(9) $$\tau =\left(F\left(X\right)=W_{Q(X)}\right)\Rightarrow \left\{\begin{array}{cc} Q=R^{m}\to N & \\ W\in R^{N} \end{array}\right.$$

A mapping relationship between an example and the associated leaf node is provided by *Q*, which stands for each regression tree’s structure. *W* stands for the leaf weight. *N* represents how many leaf nodes there are in the tree. Each $F_{n}$ is equivalent to a distinct tree structure *Q* with leaf weights *W*. When training the model, the objective is to have a good generalization capability and to have the predicted value near the real value. Considering Equation (10)

(10) $$\Omega \left(F\right)=\sum \lambda N+\frac{1}{2}\xi \sqrt{\left\|W\right\|},$$

when the training error differs from the anticipated value and assuming the loss function Ω represents the actual value in Equation (10), the regularization term’s value should be as small as possible to increase the model’s capacity for generalization. ξ and λ are used to regulate the number of leaf nodes *N* and leaf weights *W*, respectively, to avoid overfitting scenarios in Equation (10).

The loss function and the regularization period are the two elements of the objective function in Equation (11).

(11) $$\mu =\sum_{1}^{N}\eta \left(RUL_{z},{R\hat{U}L}_{z}\right)+\sum_{1}^{n}\Omega \left(F_{n}\right)$$

Because the parameters include functions, the tree ensemble model in Equation (10) cannot be optimized utilizing conventional optimization approaches in a Euclidean space. A new tree is therefore created during training to accommodate the residuals from the previous round. Consequently, for completing the model, has used *t* trees as shown in Equation (12). Thus, the model is trained to employ an additive training approach and the prediction for the *t*.

(12) $${R\hat{U}L}^{\left(t\right)}={R\hat{U}L}^{\left(t-1\right)}+F_{n}\left(X_{z}\right)$$

The following function is produced when Equation (10) is substituted into the objective function Equation (11):

(13) $$\mu^{t}=\sum_{1}^{N}\eta \left(RUL_{z},{R\hat{U}L_{z}}^{t-1}+F_{z}\left(X_{z}\right)\right)+\Omega \left(F_{t}\right)$$

The iteration of the tree model is converted into the iteration of the leaf nodes; the definitive objective function is created by replacing the optimum value with the objective function as shown in Equation (14):

(14) $$OBJ=-\left(1/2\right)\left(\sum_{1}^{K}\sqrt{G_{k}}/H_{k}+\delta \right)+\gamma K$$

The objective function is minimized as the basis for the XGBoost optimization criterion when the objective function is minimal, and the actual value is close to the predicted value.

## 4. Dataset Description

The NASA Prognostics Centre of Excellence Data Repository dataset was used to prepare the battery data. When creating the dataset to train using the recommended technique, many important battery-performing profile components were considered, including voltage, current, temperature, and capacity.

### Lithium-Ion Battery Dataset

We utilized the three operating profiles for charging, discharging, and impedance at room temperature from the NASA Prognostics Center of Excellence Data Repository’s lithium battery datasets [68]. Four lithium-ion batteries (5,6,7, and 18) were used to collect experimental lithium-ion battery data. The batteries all started off with a capacity of 2 Ah, and they were designed to reach the EoL when the capacity dropped to 70% of its starting value through a cycle of charging and discharging. Each battery underwent tests utilizing the three various charging, discharging, and impedance modes illustrated in Table 2. They were charged and discharged at room temperature (24 °C), and the impedance was measured under various working circumstances.

**Figure 4:**
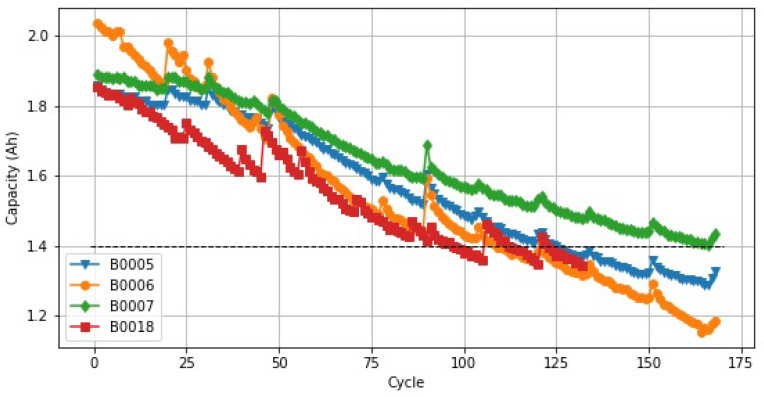
The capacity degradation process (overcharging/discharging cycle) of batteries.

There were two stages to the charging procedure, which were as follows. The first part of the charging process was a constant current charging with 1.5 A for the lithium-ion battery charge until the battery voltage reached 4.2 V. In the second part, the battery voltage was maintained at 4.2 V, and the current was decreased to 20 mA in the second stage using constant voltage charging. The discharge process was carried out at a constant current of 2 A until the voltages of batteries 5, 6, 7, and 18 reached, respectively, 2.7 V, 2.5 V, 2.2 V, and 2.5 V. Utilizing data from battery B0018 to evaluate the prediction issue validated the XGBoost method. The model was trained using the data from batteries B0005, B0006, and B0007. Figure 4 displays the capacity degradation process curve per battery and the battery failure threshold. Generally, as the number of cycles increased, the battery capacity was reduced, but the capacity regeneration phenomenon caused a local rebound in capacity after the battery had rested. Considering Figure 4, the paper used 72% of the initial capacity as the failure threshold for the B0007 battery to reach its end of life (EoL) because it was also evident that the B0007 battery did not reach the failure threshold throughout its entire life cycle. The B0007 battery’s failure threshold was not reached over its whole life cycle, as shown in Figure 4. Because it was clear during the whole life cycle that the B0007 battery did not achieve the failure threshold, the study utilized 72% of the initial capacity as the failure criterion.

**Figure 5:**
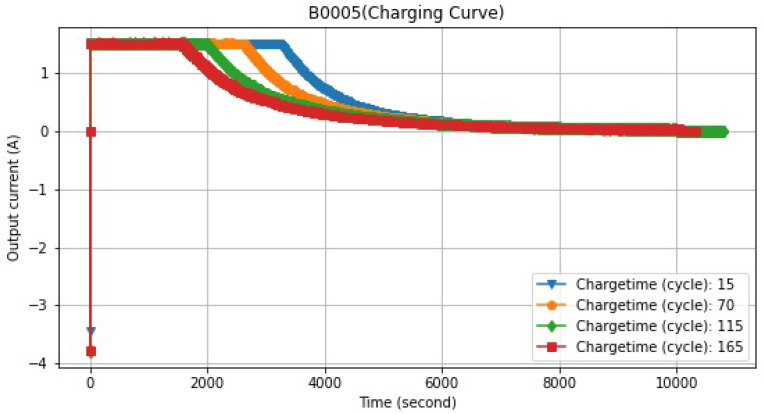
Currents during different cycles.

The data of the battery’s output current over time and several charge cycles are shown in Figure 5.

**Figure 6:**
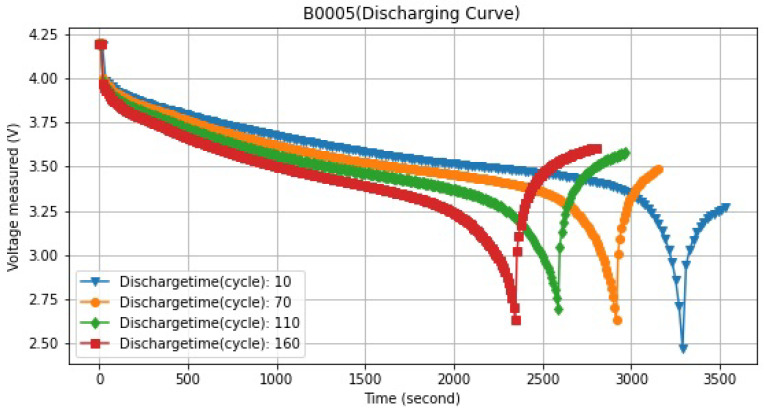
Voltage measured with different cycles.

Lithium-ion charges were dispersed erratically throughout the surface particles of the lithium-ion battery during the charging and discharging process. The number of battery particles affected increased with the size of the unevenness, shortening the battery’s life. Throughout the charging and draining procedures, the data must be described. It becomes challenging to get internal parameters due to the current’s quick fluctuation during discharge. However, because charging follows pre-established procedures, electrical characteristics are easily measured during the process. The battery parameters are taken from the charging profile to implement this suggested strategy. When the voltage is measured during the repeated discharge process, the connection between time and the measuring current differences in the data is established. The voltage, current, and temperature curves were utilized to charge and discharge the lithium-ion batteries and determine their capacity. Figure 6 displays the discharge process voltage of lithium-ion batteries with various cycles. The aged battery (with more charge–discharge cycles) reached the voltage mark of 4.2 V much sooner than new batteries, according to Figure 6.

**Figure 7:**
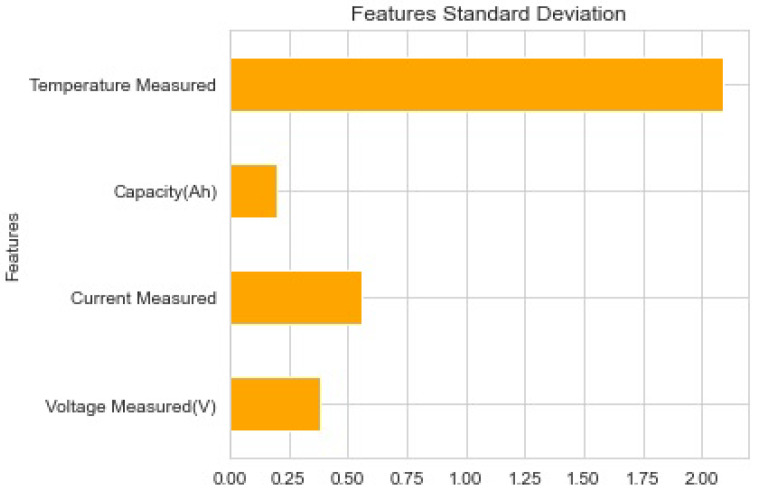
Features’ standard deviation.

Figure 7 shows that the characteristics listed above were chosen based on the feature priority indication. The battery dataset was employed for testing, training, and validation. The dataset was split into training, validation, and testing categories. The training set contained 20% of data for validation and 80% for training.

## 5. Result and Discussion

Under the same model, operating conditions, and environment, we anticipated that the model trained by one or more batteries would be capable of accurately predicting the RUL of the same battery and the RUL of other batteries in real-world scenarios. In this part, we present the experimental findings for the cases of battery test sets B0005, B0006, B0007, and B00018, respectively. This section’s primary focus is the life cycle forecasting method of the recommended solution, which also addresses the real features’ capacity degradation. Numerous case studies and experimental data sets on capacity degradation published by NASA were evaluated to verify the suggested strategy’s effectiveness and reliability.

### 5.1. Battery RUL Prediction

**Figure 8:**
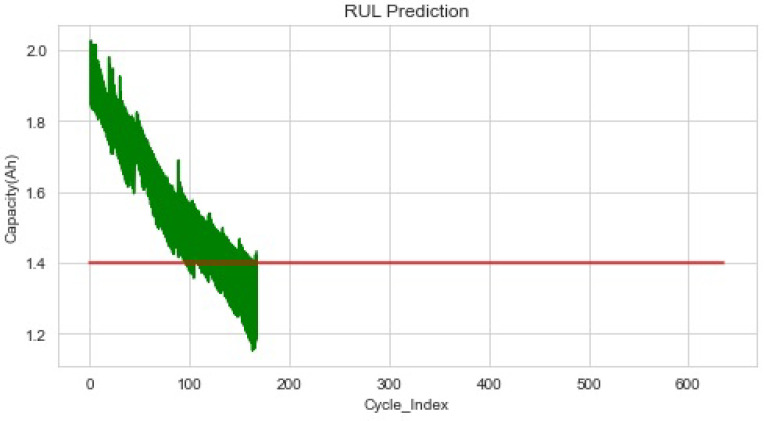
Results of the battery dataset’s prediction.

The batteries B0005, B0006, B0007, and B0018 in the NASA dataset all exhibited the same stages of capacity deterioration and decreasing trends and oscillations. The lithium-ion battery’s capacity threshold in this investigation was set at 1.4 Ah. With repeated charging and discharging cycles while the battery is in use, the internal side reactions result in performance deterioration in the battery’s capacity and internal resistance. The battery life expires once performance drops below a predetermined failure threshold (EoL). The typical failure threshold is between 70% and 80% of the rated capacity. To verify the accuracy of the recommended model for the RUL prediction, the PF can use a group of random particles to approximate the posterior probability distribution of the system state. In this study, the RUL was predicted using the parameter values that the particles represented. All of the effective particles in the particle collection were extracted. Real-time battery health state indicators were the foundation of the RUL prediction. The SOH monitoring technique was stopped, and the parameters for the present battery’s health state were recorded when the RUL threshold requirement was satisfied. Before predicting the battery capacity, the impedance was predicted to give a measurement for the RUL prediction model. The RUL prediction model used in this case was the XGBoost model, and features acquired using the aforementioned technique were utilized as inputs for model training. The suggested approach had a good prediction impact on the battery’s RUL, as shown in Figure 8.

**Figure 9:**
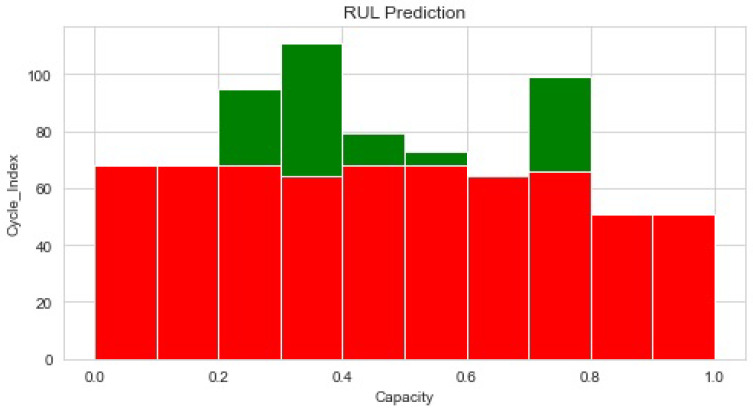
The normalized histogram capacity and the cycle of RUL prediction.

Figure 9 shows the normalized capacity and cycle of the RUL prediction through histograms of the RUL predicted by the proposed method. According to experiment findings, the suggested technique could successfully predict RUL, which could help with condition-based maintenance optimization.

### 5.2. The Parameters of XGBoost

The parameters values selected in the experimental model settings for the model are shown in Table 3.

### 5.3. Evaluation Index

The mean absolute error (MAE), the root-mean-square error (RMSE), and the following assessment criteria were used to analyze the prediction accuracy of the suggested approach, as explained in Equations (15) and (16).

(15) $$RUL_{RMSE}=\sqrt{\frac{1}{N}\sum_{n=1}^{N}{\left(RUL_{n}-{R\hat{U}L}_{n}\right)}^{2}}$$

(16) $$RUL_{MAE}=\frac{1}{N}\sum_{n=1}^{N}\left|RUL_{n}-R\hat{U}L_{n}\right|$$

The results shown in Table 4 present the estimation effect for the PF algorithm, PF-XGBoost algorithm, and improved PF-XGBoost algorithm for RUL estimation. The proposed method’s performance outperformed the other techniques based on the error rates for the RMSE and MAE. In [69], the convolution and extended short-term memory hybrid deep neural networks with different features and the dataset battery were also different. In [70], the authors used a classification, and regression tree (CART)-based XGBoost model with different features. Considering the RMSE and MAE, our proposed method had a good performance compared to these models.

**Figure 10:**
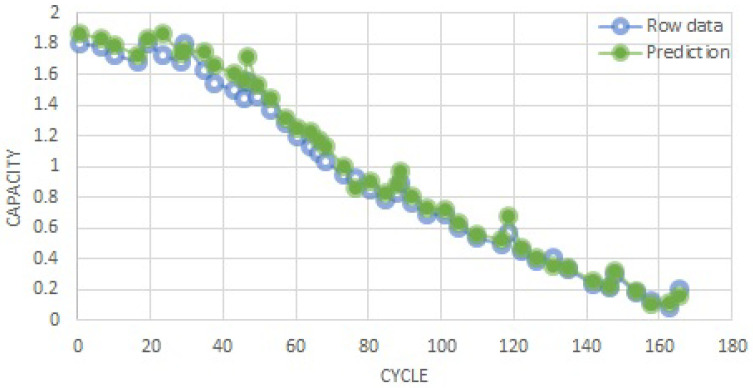
RUL estimation results.

In Figure 10, the blue and green lines depict the raw data and prediction of the RUL. The experimental results show that the XGBoost provided a high prediction performance for the batteries dataset, at least in the same operating circumstances.

## 6. Conclusions

The RUL estimate for lithium-ion batteries is a problem that was considered in this paper. The RUL’s uncertainty expression based on particle filtering could prevent situations where the anticipated value is extreme, thus ensuring the battery’s safety during usage. The RUL and KFPF of lithium-ion batteries were predicted by the XGBoost model, taking data dependency and accuracy into account. The mapping link between the input characteristics and the battery’s RUL was established using XGBoost. To get a more accurate and reliable result when estimating the RUL, a KF was utilized to adjust the RUL value calculated by XGBoost. Experimental findings showed that this technique could choose relevant traits and make accurate predictions. The suggested model’s MAE and RMSE were 0.0179% and 0.0173%, respectively. The model performed well and had improved prediction accuracy. The proposed method can effectively improve the prediction accuracy of the RUL of a lithium-ion battery.

## 7. Future Work

Exploring physical models for various battery types and integrating data-driven methodologies with more complex physical-based battery models are among the tasks to be done in the future concerning the RUL prediction. Another task that can also be further improved is the accuracy of the battery’s SOH prediction model, which is thought to be more accurate due to the battery’s involved operational environment elements. There are dangers associated with operating batteries, and the maker of the battery product should set up an appropriate scientific risk assessment and mitigation strategy. Additionally, several clever algorithms can be used in follow-up research to obtain an accurate long-term prediction.

## Figures and Tables

**Table 1:** Comparison of different RUL prediction techniques.

Author	Proposed Approach	Type	Benefit
Ungurean et al. [57]	Gated recurrent unit neural network	AI-based	Fast, simple input
Tao et al. [58]	Support vector regression	AI-based	Easy implementation, easily updated, robust to outliers
Li et al. [59]	Convolutional neural network	AI-based	Cost and time remain on top, high quality and accuracy
Fan et al. [60]	Gaussian process regression	Stochastic-based	Ability to provide uncertainty measurements
Xu et al. [61]	Wiener process	Stochastic-based	Instrument errors in filtering theory and disturbances
Wu et al. [62]	Neural network and bat-based particle filter	Hybrid methods	Optimization, improved accuracy
Gou et al. [63]	Hybrid ensemble data-driven	Hybrid methods	Improved accuracy, increased stability and effectiveness

**Table 2:** Experimental specifications for the battery dataset.

Battery Number	Charging Constant Current (A)	Charging Charge Cut-Off Voltage (V)	Discharging Constant Current (A)	Discharge Cutoff (V)	Operating Temperature
B_5	1.5	4.2	2.0	2.7	24 °C
B_6	1.5	4.2	2.0	2.5	24 °C
B_7	1.5	4.2	2.0	2.2	24 °C
B_18	1.5	4.2	2.0	2.5	24 °C

**Table 3:** Parameters values utilized in the experimental model.

Sr	Parameter	XGBoost
1	n_estimators	300
2	max_depth	8
3	min_child_weight	0.01
4	n_jobs	12
5	random_state	42
6	verbosity	None
7	base_score	0.5
8	booster	gbtree

**Table 4:** Comparison of error rates between previous approaches and the proposed method.

Ref-No	Method	RMSE	MAE
[69]	Convolution and long short-term memory hybrid deep neural networks	16.12%	13.26%
[70]	XCART (classification and regression tree based extreme gradient boosting)	0.0262%	0.0184%
Proposed method	XGBoost	0.0179%	0.0173%

## Data Availability

Not Available.
